# A Very Rare Case of Brucellosis-Related Tubo-ovarian Abscess

**DOI:** 10.1590/0037-8682-0501-2019

**Published:** 2020-02-07

**Authors:** Handan Alay, Fatma Kesmez Can, Emsal Pınar Topdağı Yılmaz

**Affiliations:** 1 Department of Infectious Diseases and Clinical Microbiology, Faculty of Medicine, Ataturk University, Erzurum, Turkey.; 2 Department of Obstetrics and Gynecology, Faculty of Medicine, Ataturk University, Erzurum, Turkey.

**FIGURE 1:**
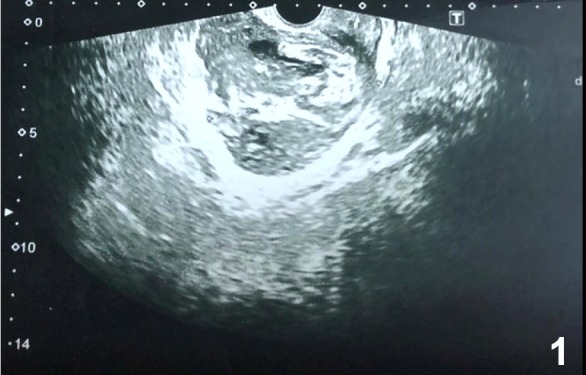
Pelvic ultrasonography showing an 8 cm diameter abscess.

**FIGURE 2:**
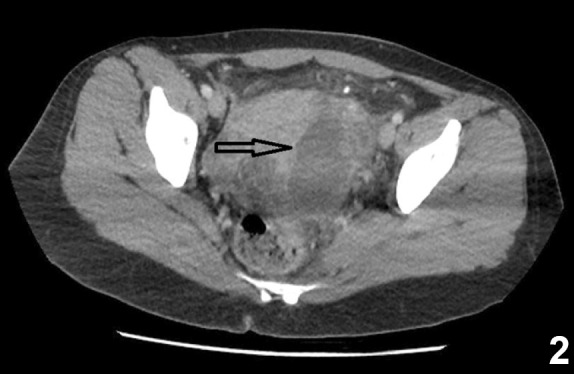
Abdomino-pelvic computed tomography showing a 69×41 mm abscess adjacent to the iliac vascular structures.

**FIGURE 3:**
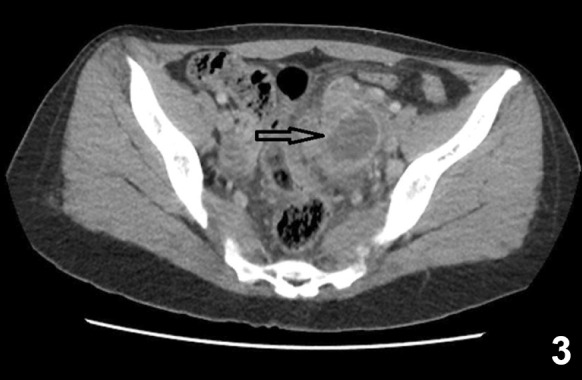
Shrinkage of the abscess on the 14^th^ day of brucellosis treatment.

A 30-year-old patient presented with persistent left inguinal pain, abdominal pain, and fever for 10 days. Abdominal examination revealed hepatomegaly, splenomegaly, and left lower quadrant and suprapubic region tenderness. An 8 cm diameter abscess was observed in abdominal ultrasonography (Figure 1). Pelvic computed tomography revealed a 69×41 mm abscess with intense inflammation in the surrounding mesenteric area extended up to the iliac vasculature (Figure 2). Brucella spp. was detected in a blood sample. The brucella tube agglutination test was found positive at 1/640. The patient had a history of consuming unpasteurized milk and milk products. Brucellosis was diagnosed based on clinical, laboratory, and radiological findings and the patient history. The patient received doxycycline (2×200 mg), rifampicin (1×600 mg), and trimethoprim-sulfamethoxazole (2×1) therapy. The fever decreased on the third day of treatment, and the abscess contracted (33×40 mm) after 14 days (Figure 3). The gynecologist did not plan surgery due to the clinical and laboratory response and the shrinkage of abscess with treatment. 

Brucellosis, a zoonotic infection, is an important public health problem in many developing countries^1^. Tubo-ovarian abscess frequently exhibits an acute course together with genitourinary and systemic symptoms^2^. Brucellosis can affect many organs requiring a multidisciplinary approach for patient management. This etiology should be considered in differential diagnosis of patients with tubo-ovarian abscess in endemic regions.
